# Diarrhoeagenic *Escherichia coli* in mother-child Pairs in Ile-Ife, South Western Nigeria

**DOI:** 10.1186/s12879-016-1365-x

**Published:** 2016-01-25

**Authors:** Babatunde W. Odetoyin, Jennifer Hofmann, Aaron O. Aboderin, Iruka N. Okeke

**Affiliations:** 1 Department of Medical Microbiology and Parasitology, Obafemi Awolowo University, Ile-Ife, Nigeria; 2 Department of Biology, Haverford College, 370 Lancaster Avenue, Haverford, PA 19041 USA; 3 Faculty of Pharmacy, University of Ibadan, Ibadan, Nigeria

**Keywords:** Diarrhoea, Diarrhoeagenic *Escherichia coli*, Mother-child pairs, Antimicrobial resistance

## Abstract

**Background:**

Diarrhoeagenic *Escherichia coli* (DEC) pathotypes are among the most common bacterial causes of morbidity and mortality in young children. These pathogens are not sought routinely and capacity for their detection is limited in Africa. We investigated the distribution and dissemination of DEC in 126 children paired with their mothers in a Nigerian community.

**Methods:**

A total of 861 *E. coli* were isolated from 126 children with diarrhoea and their mothers. Antimicrobial susceptibility of each isolate was determined by Kirby-Bauer disc diffusion technique. All the isolates were screened for DEC markers by multiplex PCR. Genetic relatedness of DEC strains was determined by flagellin typing and Insertion element 3 (IS*3*)-based PCR.

**Results:**

DEC were identified from 35.7 % of individuals with the most common pathotype being shiga toxin-producing *E. coli* (42, 16.7 %). Identical pathotypes were found in 13 (10.3 %) of the mother-child pairs and in three of these strains from mothers and their children showed identical genetic signatures. Over 90 % of DEC isolates were resistant to ampicillin, sulphonamide, tetracycline, streptomycin or trimethoprim, but only 9 (7.2 %) were ciprofloxacin resistant

**Conclusion:**

The data suggest that healthy mothers are asymptomatic reservoirs of multiply-resistant strains that are pathogenic in their children and there are instances in which identical strains are found in mother-child pairs.

## Background

Each year, diarrhoeal disease is responsible for deaths of one in every ten children less than 5 years, resulting in 750,000 fatalities worldwide especially in sub-Saharan Africa and south Asia [1, 2]. In some parts of these regions, mortality rates are reducing considerably by about 4 % every year due to improved hygiene practices and the success of oral rehydration therapy. Morbidity due to diarrhoea is still however high in many other countries where outbreaks of diarrhoeal diseases continue to affect many infants and young children [1, 3, 4]. Thus, understanding the epidemiology and transmission of the disease is important to accelerating the decline in mortality and morbidity.

Thirty to forty percent of cases of acute diarrhoea in children below 5 years of age are caused by diarrhoeagenic *Escherichia coli* (DEC) [5–7]. Six pathotypes of DEC are recognized on the basis of specific virulence factors. These are enterotoxigenic *E. coli* (ETEC), enteroinvasive *E. coli* (EIEC), enteropathogenic *E. coli* (EPEC), enterohemorrhagic *E. coli* (EHEC) (including shiga toxin producing *E. coli* (STEC)), enteroaggregative *E. coli* (EAEC), and diffusely adherent *E. coli* (DAEC) [8]. The epidemiological significance and prevalence of each category of DEC in childhood diarrhoea varies from one geographical location to another. In spite of these variations, very few studies with discriminatory methodology have been carried out in sub-Saharan Africa to investigate the burden of the pathogens [7–12].

Diarrhoea is spread primarily through faecal-oral route, especially when contaminated food or water is consumed, though evidence also showed that secondary transmission or person-to-person transmission of diarrhoeal pathogens also occurs, particularly among members of the same household. If sharing of strains within the same household contributes significantly to the perpetuation of virulent clones, efforts to prevent within-household transmission could truncate the spread of diarrhoeagenic *E. coli* clones in the community, in addition to protecting vulnerable children from infection [13, 14].

We sought to evaluate the occurrence of DEC in children with diarrhoea as well as the role of close contact in intra-familial co-occurrence of DEC by comparing DEC recovery from these children and their mothers in a Nigerian community.

## Methods

### Study population and sample collection

Approval (IEC No. 00005422) for the study was obtained from the Ethics and Research committee of Obafemi Awolowo University Teaching Hospitals Complex, Ile-Ife. Participants were recruited at the State Hospital Oke-Ogbo, Ile-Ife, Osun State, between February 2008 and February 2011. All the participants gave informed consent. One hundred and twenty-six children aged five and below with diarrhoea of not more than 2 weeks duration, paired with their mothers (i.e. 252 individuals), were included. Consenting mothers and their children were requested to produce stool samples in sterile universal bottles. All samples were transported within 2 hours of collection to the laboratory for processing.

### Detection and Identification of *Escherichia coli* strains

Faecal samples were inoculated onto Eosin Methylene Blue (EMB) agar plates (Oxoid Ltd., Basingstoke, Hampshire, England) and incubated for 24 h aerobically at 37 °C. Up to five morphologically distinct colonies typical of *E. coli* were picked and identified by standard biochemical testing [15].

### Antimicrobial susceptibility testing

Antimicrobial susceptibility testing for the isolates was performed by the Kirby-Bauer disc diffusion technique on Mueller Hinton agar (CM0337) (Oxoid Ltd., Basingstoke, Hampshire, England). Antibiotics tested included ampicillin (AMP) (l0μg), streptomycin (STREP) (l0μg), ciprofloxacin (CIP) (5 μg), nalixidic acid (NAL) (30 μg), tetracycline (TET) (30 μg), chloramphenicol (CHLO) (30 μg), sulphonamide (SUL) (300 μg), and trimethoprim (TRIM) (5 μg) (Remel, U.S.A). The inoculated plates were incubated at 37 °C for 24 h. Interpretation of the diameters of the zones of inhibition was done according to the guidelines set by clinical laboratory and standard institute (2008). *E. coli* ATCC 25922 was used as the quality control strain.

### DNA Extraction

Isolate stocks were produced by sub-culturing single colonies from primary isolation plates. Two to three colonies of the isolates were grown overnight in 5 ml of peptone broth (Oxoid). A 1 ml aliquot of the culture was centrifuged at 10,000 rpm for 2 min in a microcentrifuge (Biorad, USA). The cell pellet was suspended in 100 μl of sterile water and boiled for 10 min. The resulting DNA suspension was used as template DNA in subsequent polymerase chain reaction (PCR) amplifications.

### Screening for Diarrhoeagenic *E. coli*

*E. coli* isolates were screened for virulence genes that characterized five different pathotypes of diarrhoeagenic *E. coli* namely ETEC, EIEC, EPEC, EAEC, and EHEC including STEC by multiplex PCR [16] (Table 1). *E. coli* strains E2348/69, 042, H10407, EDL933, and E137 served as positive controls for EPEC, EAEC, ETEC, EHEC (STEC), and EIEC respectively. *E. coli* DH5α, which lacks all the diarrhoeagenic genes was used as a negative control. Amplified reactions (5 μl) were electrophoresed on a 1.5 % (w/v) agarose gel in 1X TAE buffer and visualized in ultraviolet light after ethidium bromide staining.

**Table 1:** Polymerase chain reaction primers for diarrhoeagenic genes, IS*3* and *fli*C of *Escherichia coli* ^a^

Target gene or probe	Type	Primer Designation	Primers (5’ to 3’)	Amplicon size (base pair)
*eae*	EPEC/EHEC	eae 1	CTGAACGGCGATTACGCGAA	917
		eae 2	CCAGACGATACGATCCAG	
*bfp*	EPEC	bfp 1	AATGGGCTTGCGCTTCCAG	326
		bfp 2	GCCGCTTTATCCAACCTGGTA	
CVD432	EAEC	EAEC1	CTGGCGAAAGACTGTATCAT	630
		EAEC2	CAATGTATAGAAATCCGCTGTT	
LT gene	ETEC	LTf	GGCGACAGATTATACCGTGC	450
		LTr	CGGTCTCTATTATACCGTGC	
ST gene	ETEC	STf	ATTTTTMTTTCTGTATTRTCTT	190
		STr	CACCCGGTACARGCAGGATT	
*ipaH*	EIEC	IpaH1	GTTCCTTGACCGCCTTTCCGATACCGTC	600
		IpaH2	GCCGGTCAGCCACCCTCTGAGAGTAC	
*Stx*1	EHEC/STEC	Stx1f	ATAAATCGCCATTCGTTGACTAC	180
		Stx2r	AGAACGCCCACTGAGATCATCC	
*stx*2	EHEC/STEC	Stx2f	GGCACTGTCTGAAACTGCTCC	255
		Stx2r	TCGCCAGTTATCTGACATTCTG	
IS3	N/A	IS3	ACACTTAGCCGCGTGTCC	Variable
*fliC*	N/A	F-FLIC1	ATGGCACAAGTCATTAATACCCAAC	Variable
		R-FLIC2	CTAACCCTGCAGCAGAGACA	

^a^ *eae* intimin, *EPEC* Enteropathogenic *E. coli*, *EHEC* Enterohemorrhagic *E. coli*, *bfp* bundle forming pilus, *CVD432* aggregative adherence plasmid marker, *EAEC* Enteroaggregative *E. coli*, *LT* Heat-labile toxin, *ETEC* Enterotoxigenic *E. coli*, ST Heat-stable toxin, *ipaH* invasive plasmid antigen, *EIEC* Enteroinvasive *E. coli*, *stx* shiga toxin, *STEC* shiga toxin producing *E. coli*, *N/A* Not applicable

### Genetic profiling

Insertion element 3 (IS*3*)-based PCR profiles were generated by amplification with the IS3A primer of Thompson et al. [17] (Table 1) and electrophoresis on 1.5 % agarose gels as previously described [18]. PCR-Restriction fragment length polymorphisms of the *fliC* gene were performed by amplifying the *fliC* allele and digesting amplicons with *Rsa*I, as described by Fields et al. [19]. Each distinct PCR-RFLP profile was assigned a letter code.

### Statistical analysis

The Chi square (*χ*2) and Fischer’s exact test (two tailed), performed using SPSS statistical software (version 15) (Chicago, IL, USA) were used to determine the statistical significance of the data. All reported *p*-values were two-sided and a *p*-value of less than or equal to 0.05 was considered to be statistically significant.

## Results

### Subjects and Faecal *Escherichia coli* isolates

A total of 252 stool samples were collected from 126 children with diarrhoea aged one month to five years (13.57 ± 11.35) paired with their apparently healthy mothers aged 15 to 46 years (19.74 ± 10.88). The children were distributed as: 0–6 months 25 (19.8 %), 7–12 months 57 (45.2 %), 13–24 months 33 (26.2 %), and 25–60 months 11 (8.7 %). From the 252 samples examined, 861 strains of *E. coli* comprising of 438 from children and 423 from mothers were isolated.

### Diarrhoeagenic *Escherichia coli* pathotypes

Of 252 stool samples examined, 90 (35.7 %), comprised of 45 samples from mothers and 45 from children were positive for at least one pathotype of DEC. In all, there were 125 different DEC strains identified of which 65 (25 %) and 60 (48 %) were from children and mothers respectively (Table 2). Shiga toxin producing *E. coli* (STEC) 42 (16.7 %) was the most prevalent sub-pathotype of DEC detected. Thirty-two of these STEC strains harbored both *stx*1 and *stx*2 genes. Other pathotypes identified were enterotoxigenic *E coli* (ETEC) 40 (15.9 %), enteroaggregative *E. coli* 21 (8.3 %), and enteropathogenic *E. coli* (EPEC) 13 (5.2 %). Enteroinvasive (EIEC) 2 (0.8 %) were found only among isolates from mothers. Among the ETEC strains, heat-labile toxin gene alone was more commonly found, 23 (57.5 %) than the heat stable toxin gene 16 (40 %), whereas only 1 of 40 (2.5 %) possessed both genes.

**Table 2:** Distribution of diarrhoeagenic virulence genes in faecal *Escherichia coli* from mother and child pairs*

	No. (%) of subjects		
DEC Type	Mothers	Children	*P*-value (*χ* ^2^)
	(*n* = 126)	(*n* = 126)	
EAEC	11 (8.7)	10 (8.7)	1.000
STEC	18 (14.3)	24 (19)	0.310
*stx1* (only)	6 (4.8)	4 (3.2)	0.749
*stx2* (only)	-	-	-
*stx1*and *stx2*	12 (9.5)	20 (15.9)	0.130
EPEC	7 (5.6)	7 (5.6)	0.776
Typical (*bfp*+)	7 (5.6)	3 (2.4)	0.334
Atypical (*bfp*-)	0 (0)	4 (3.2)	0.122
ETEC	20 (15.9)	20 (15.9)	1.000
LT (only)	9 (7.1)	14 (11.1)	0.274
ST (only)	10 (8.7)	6 (4.8)	0.310
LT and ST	1 (0.8)	0 (0)	1.000
EIEC	2 (1.6)	0 (0)	0.498
Total	60 (48)	65 (52)	0.527

*p-value Probability value of chi square, *EAEC* Enteroaggregative *E. coli*, *EHEC* Enteroheamorrhagic *E. coli*, *STEC* Shiga toxin producing *E. coli*, stx shiga toxin, *EPEC* Enteropathogenic *E .coli*, bfp bundle forming pilus, *ETEC* Enterotoxigenic *E. coli*, *LT* Labile toxin, *ST* Stable toxin, *EIEC* Enteroinvasive *E. coli*

Multiple DEC pathotypes were recovered from 8 (6.3 %) children and 10 (7.9 %) mothers. As shown in Table 3, the most frequent of such combinations was STEC with EPEC, 3 (16.7 %).

**Table 3:** Frequency of multiple pathotypes of diarrhoeagenic *Escherichia coli* among the mother and child groups^a^

Multiple pathotypes	No (%) of positive samples	
	Mother (*n* = 126)	Child (*n* = 126)
ETEC (ST) + STEC	1 (0.8)	1 (0.8)
ETEC (LT) + STEC	1 (0.8)	0 (0)
ETEC (ST) + ETEC (LT)	2 (0.8)	0 (0)
EPEC + ETEC(ST)	1 (0.8)	1 (0.8)
EPEC + ETEC(LT) + ETEC(ST)	0 (0)	1 (0.8)
EHEC + EPEC + ETEC(LT) + EAEC	0 (0)	1 (0.8)
EAEC + ETEC(LT + ST)	1 (0.8)	0 (0)
EAEC + ETEC(LT)	0 (0)	1 (0.8)
EAEC + ETEC(ST) + EIEC	1 (0.8)	0 (0)
EPEC + STEC	2 (1.6)	1 (0.8)
EHEC + STEC	0 (0)	1 (0.8)
EAEC + STEC	1 (0.8)	1 (0.8)
Total	10 (7.9)	8 (6.3)

^a^ *ETEC* Enterotoxigenic *E. coli*, *ST* heat stable toxin, *STEC* shiga toxin producing *E. coli*, *LT* heat labile toxin, *EPEC* Enteropathogenic *E. coli*, *EHEC* Enteroheamorrhagic *E. coli*, *EAEC* Enteroaggregative *E. coli*, *EIEC* Enteroinvasive *E. coli*

As shown in Table 4, DEC were most commonly recovered from children aged between age 7 and 24 months. Recovery within the first 6-months of life and after two years was relatively rare. STEC was isolated across the different age categories, whereas EPEC was most commonly seen in children less than 1 year. The other pathotypes were most commonly recovered from children 7–24 months.

**Table 4:** Frequency and age distribution of diarrhoeagenic *Escherichia coli* isolated from faecal samples of children with diarrhoea^a^

Age (mths)	No of Children (126)	Total isolates (65)	Types of Diarrhoeagenic *E. coli*				
			EPEC (*n* = 7)	EAEC (*n* = 10)	ETEC (*n* = 20)	STEC (*n* = 24)	EHEC (*n* = 4)
0–6	25	8 (32)	2 (8)	0 (0)	1 (4)	5 (20)	0 (0)
7–12	57	32 (56.1)	4 (7)	4 (7)	11 (19.3)	10 (17.5)	3 (5.3)
13–24	33	23 (69.7)	1 (3)	5 (15.2)	8 (24.2)	8 (24.2)	1 (3)
25–60	11	2 (18.2)	0 (0)	1 (9.1)	0 (0)	1 (9.1)	0 (0)

^a^ *Age* Age categories of children, *EPEC* Enteropathogenic *E. coli*, *EAEC* Enteroaggregative *E. coli*, *ETEC* Enterotoxigenic *E. coli*, *STEC* Shiga toxin producing *E. coli*, *EHEC* Enteroheamorrhagic *E. coli*

### Recovery of identical pathotypes of DEC from mother and child pairs

Identical DEC pathotypes were detected in 13 (10.3 %) of all the 126 mother-child pairs tested. These consisted of five ETEC, five EAEC and three STEC strains. EAEC was recovered in 2 (1.6 %) of children (with infected mothers) in the age groups 7–12 months and 13–24 months while 1 (0.8 %) was recovered in age group 25–60 months. No EAEC was detected in children below 7 months of age. Four of five pairs from which ETEC was recovered included infected children in the age range 7–12 months. No EPEC and EHEC were recovered from children with colonized mothers (Table 5).

**Table 5:** Age distribution of mother and child pairs with identical diarrhoeagenic *E. coli* pathotypes^a^

DEC Types	Paired	Age in months			
	Mother-child				
	*N* = 126	0–6 (%)	7–12 (%)	13–24 (%)	25–60 (%)
ETEC	5 (4.0)	0 (0)	4 (3.2)	1 (0.8)	0 (0)
EAEC	5 (4.0)	0 (0)	2 (1.6)	2 (1.6)	1 (0.8)
STEC	3 (2.4)	0 (0)	3 (2.4)	0 (0)	0 (0)
Total	13 (10.3)	0 (0)	9 (7.2)	3 (2.4)	1 (0.8)

^a^ *ETEC* Enterotoxigenic *E. coli*, *EAEC* Enteroaggregative *E. coli*, *STEC* Shiga toxin producing *E. coli*

**Fig. 1:**
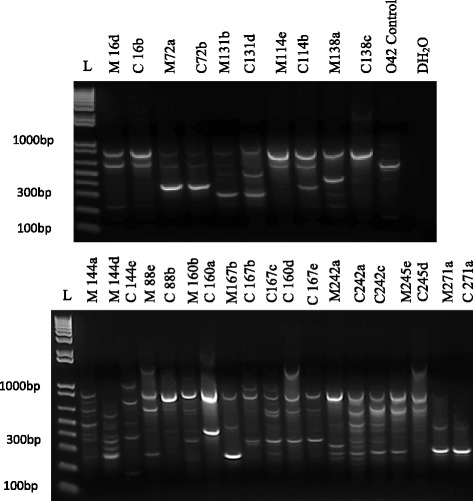
IS3 profiling gels of isolates with similar pathotypes of *Escherichia coli* from mother and child pairs. Lanes L: 1 kb ladder plus

**Fig. 2:**
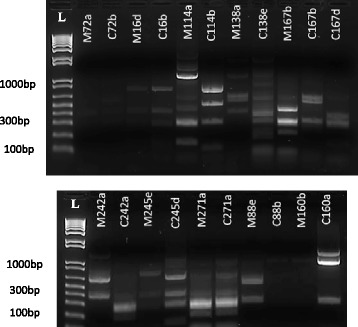
*Rsa*I RLFP profiles from DEC isolates with identical or similar IS3 profiles. Lanes L: 1 kb ladder plus

We determined whether isolates of the same pathotype obtained from mother-infant pairs produced identical or similar band patterns after PCR profiling with an IS*3* primer (Fig. 1). Isolates showing non-identical profiles that were similar were shortlisted because in our experience, IS*3* profiles can vary slightly between experiments. Five within-pair similarities were seen: these strains showed identical or similar (not more than two different bands) profiles. Isolates showing within-pair similarities were then subjected to *fliC* allele typing by PCR-RFLP. Based on these profiles indistinguishable isolates were obtained from five mother-infant pairs (Table 6). Three isolates pairs showed identical/similar IS*3* profiles and identical *fliC* PCR-RFLP patterns. Two of these pairs were EAEC (C16b and M16d, C271a and M271a) and the third STEC isolates (C72b and M72a). Two ETEC isolates showed similar/identical IS*3* patterns and produced no *fliC* amplicons (Table 6). Within-pair IS3 profiles were distinctly different for seven pairs and in the case of one ETEC pair, although the IS*3* profiles were identical, *fliC* PCR-RFLP patterns were different (Fig. 1, Table 6). Thus we conclude that in the case of three to five mother infant pairs, identical DEC isolates were recovered from mother and child. IS*3* profiles (Fig. 1) and *fliC* PCR-RFLP patterns (Fig. 2) differed considerably among different pairs suggesting that a diverse range of DEC strains are circulating in the study population.

**Table 6:** Isolates from mother-child pairs demonstrating the same pathotype and similar IS*3* profiles^a^

DEC Types	Isolate No	Resistance profiles	*fli*C	IS3
ETEC	C114b	TET SUL AMP	NA	G
	M114e	TET TRIM SUL AMP NAL CHLO	NA	G
	C131d	TET TRIM SUL AMP STREP NAL CHLO	NA	F
	M131b	TET TRIM SUL AMP STREP NAL	NA	F
	C138c	TET TRIM SUL AMP STREP	S	E
	M138a	TET TRIM SUL AMP CIP STREP NAL CHLO	T	E
EAEC	C16b	TET TRIM SUL AMP STREP CHLO	R	B
	M16d	TET TRIM SUL AMP STREP NAL CHLO	R	B
	C271a	TET TRIM SUL AMP STREP NAL CHLO	P	C
	M271a	TET TRIM SUL AMP STREP CHLO	P	C
STEC	C72b	TET TRIM SUL AMP STREP NAL	Q	D
	M72a	TET TRIM SUL AMP STREP	Q	D

^a^ *EAEC* Enteroaggregative *E. coli*, *ETEC* Enterotoxigenic *E. coli*, *STEC* Shiga toxin producing *E. coli*, *C* child, *M* mother, *TET* Tetracycline, *TRIM* Trimethoprim, *SUL* Sulphonamide, *AMP* Ampicillin, *CIP* Ciprofloxacin, *STREP* Streptomycin, *NAL* Nalidixic acid, *CHLO* Chloramphenicol, *IS* Insertion sequence, *NA* No amplicon

### Antimicrobial resistance patterns of diarrhoeagenic *Escherichia coli* (DEC) isolates and Non Diarrhoeagenic *Escherichia coli* (non-DEC) from mother and child pairs

The DEC (*n* = 125) isolates exhibited high rates of resistance to most antibacterial commonly available and that were also tested in the study. These included ampicillin 121 (96.8 %), sulphonamide 118 (94.4 %), tetracycline 119 (95.2 %), streptomycin 115 (92 %), trimethoprim 113 (90.4 %), chloramphenicol 58 (46.4 %), and nalidixic acid 58 (46.4 %). A much lower rate of resistance was seen to ciprofloxacin 9 (7.2 %). Similar profiles were seen with specific pathotypes detected. While all the EAEC (21 of 21), and EIEC (2 of 2) strains were resistant to tetracycline, sulphonamide, ampicillin and streptomycin, they were all susceptible to ciprofloxacin (Table 7).

**Table 7:** Antimicrobial resistance pattern of diarrhoeagenic *E. coli* isolates^a^

Antimicrobial agents	No of resistant DEC isolates (%)							Non DEC (%)	*P*-value
	EPEC (*N* = 14)	EAEC (*N* = 21)	EHEC (*N* = 6)	ETEC (*N* = 40)	STEC (*N* = 42)	EIEC (*N* = 2)	Total (*N* = 125)	*N* = 736	
Tetracycline	14 (100)	21 (100)	6 (100)	37 (92.5)	39 (92.9)	2 (100)	119 (95.2)	683 (92.8)	0.326
Trimethoprim	11 (78.6)	21 (100)	5 (83.3)	36 (90)	38 (90.5)	2 (100)	113 (90.4)	617 (83.8)	0.059
Sulphonamide	12 (85.7)	21 (100)	6 (100)	37 (92.5)	40 (95.2)	2 (100)	118 (94.4)	689 (93.6)	0.738
Ampicillin	13 (92.9)	21 (100)	6 (100)	39 (97.5)	40 (95.2)	2 (100)	121 (96.8)	666 (90.5)	0.061
Ciprofloxacin	1 (7.1)	0 (0)	1 (16.7)	3 (7.3)	4 (9.5)	0 (0)	9 (7.2)	65 (8.8)	0.547
Streptomycin	12 (85.7)	21 (100)	6 (100)	35 (87.5)	39 (92.9)	2 (100)	115 (92)	706 (95.9)	0.090
Nalidixic acid	4 (28.6)	8 (38.1)	3 (50)	20 (50)	23 (54.8)	1 (50)	58 (46.4)	351 (47.7)	0.902
Chloramphenicol	16 (42.9)	12 (57.2)	1 (16.7)	13 (32.5)	25 (59.5)	1 (50)	58 (46.4)	356 (48.4)	0.538

^a^ *DEC* diarrhoeagenic *E. coli*, *EPEC* Enteropathogenic *E. coli*, *EAEC* Enteroaggregative *E. coli*, *EHEC* Enteroheamorrhagic *E. coli*, *ETEC* Enterotoxigenic *E. coli*, *STEC* Shiga toxin producing *E. coli, EIEC* Enteroinvasive *E. coli*, *P* probability

When the antimicrobial resistance profiles of 125 diarrhoeagenic *E. coli* isolates were compared with that of the 736 non-DEC isolates, there was no significant difference in the rates of resistance as both groups recorded similar high rates of resistance to all the antibiotics except to ciprofloxacin (Table 7).

Multidrug resistance (i.e. resistance to three or more different classes of antibiotics) was present in 98.4 % of the DEC isolates (Table 8). The multi-resistance pattern of the DEC isolates was compared with the non-DEC isolates as shown in Table 5. Notably, 125 (98.4 %) of DEC isolates were resistant to three or more antibiotics in comparison to 736 (98.9 %) of the non-DEC isolates showing that there was no significance difference between the two groups (*χ*2 = 0.115; *p* = 0.621).

**Table 8:** Multi resistance of diarrhoeagenic *E. coli* and non diarrhoeagenic *E. coli* to eight antibiotics^a^

No of tested antibiotics	No of resistant isolates		*P*-value
	DEC *N* = 125	non-DEC *N* = 736	
0	0 (0)	0 (0)	-
1	0 (0)	3 (0.40)	1.000
2	2 (1.6)	12 (1.6)	0.980
3	10 (7.6)	9 (6.5)	0.583
4	7 (5.6)	60 (8.2)	0.325
5	30 (24)	200 (27.2)	0.458
6	46 (36.8)	207 (28.1)	0.063
7	28 (22.4)	183 (24.9)	0.554
8	6 (4.8)	38 (5.2)	0.865
≥3	123 (98.4)	728 (98.9)	0.621

^a^ *P* probability, *DEC* diarrhoeagenic *Escherichia coli*, *non-DEC* non- diarrhoeagenic *Escherichia coli*

## Discussion

Diarrhoea remains one of the main causes of morbidity and mortality in children worldwide and a large proportion of bacterial gastroenteritis is caused by diarrhoeagenic *E. coli* [1, 20]. In the present study, DEC was isolated in 90 (35.7 %) samples from 126 mother-child pairs, of which 45 (35.7 %) were from children with diarrhoea and 45 (35.7 %) were from their mothers. The prevalence of DEC from children with diarrhoea in this study is comparable to 37.1 % found in a previous controlled study among children with diarrhoea in Ile-Ife [21]. However, a limitation of the study is that children without diarrhoea were not included as controls.

DEC was most commonly recovered from children between 7 and 24 months of age. Children below six months of age may have been protected by maternal antibodies from exclusive breastfeeding while older children may be protected by their own acquired immunity following exposure to the infectious agent earlier in life. The high prevalence of DEC among children in the age group 7–24 months emphasizes the vulnerability during and following weaning, at a time when children are still in close contact with their mothers [22–25].

Multiple DEC categories were recovered from samples from 8 (6.3 %) diarrheic children between 5 and 13 months of age (Data not shown). Mixed infections are reported to involve more dehydration when compared with episodes caused by a single diarrhoeagenic *E. coli* pathotype [26, 27].

STEC 42 (16.7 %) was the most prevalent DEC pathotype isolated from both children with diarrhoea and mothers. This observed high prevalence of STEC found in this study is similar to the findings of Garcia [28] and Alikhani [29] but it is in contrast to other studies that reported very low prevalence in children with diarrhea [11, 30, 31]. The detection of STEC in this study suggests the possibility of community-wide risk of severe STEC disease such as hemorrhagic colitis and hemolytic uremic syndrome. It may also point to a high prevalence of *stx* phages and therefore the potential for horizontal transfer of virulence creating new pathogenic lineages or hybrid strains. When STEC were found with another pathotype, the second pathotype was most commonly EPEC. Because EHEC carry *eae* and shiga toxin genes, this combination might have been misinterpreted to represent EHEC had we used stool PCRs rather than the isolation and identification methods we employed.

ETEC and EAEC have been reported as important causes of childhood diarrhoea in West Africa [7, 32–35]. In this study, ETEC was detected in specimens from 20 (15.9 %) and EAEC 7.9 % of children. EAEC recovery was lower than seen in some other Nigerian studies [11, 21]. The low recovery may be attributable to the use of a DNA probe, CVD432 to detect EAEC in this study in place of HEp-2 adherence assay which is the Gold Standard for EAEC detection [21, 36]. The HEp-2 adherence assay is currently not implementable in Nigeria because tissue culture is not available in existing bacteriology labs. Recovery of identical pathotypes of ETEC and EAEC from mother and child pairs suggests the possible role of person-to-person transmission of infection of these two DEC pathotypes although infection from a common source can also not be ruled out.

Twenty-three (57.5 %) of the 40 ETEC strains identified in this study possess genes encoding the heat-labile toxin (LT) and 16 (40 %) encode ST. In the recent GEMS study and earlier research, ST-producing ETEC have shown greater association with diarrhoea than almost all other enteropathogens at multiple developing country sites [7]. It is therefore of concern that 11 (9.5 %) of mothers were found carrying this subcategory of ETEC, which could potentially be transmitted to vulnerable children. Mothers also harbored all other DEC categories recovered from the children with diarrhoea in this study, except EIEC, adding to available data that mothers and other adults may be reservoirs for enteric pathogens associated with infantile diarrhea [37]. Of note is the presence of identical DEC strains in three to five mother-infant pairs. Interestingly, we did not see identical strains among unpaired isolates, suggesting that these strains are probably not clones that are disseminated community-wide. Consequently, the data point to transmission of some strains to children by their mothers, or vice versa, or interfamilial infection from a common source. Whichever of these scenarios is in play, interventions that interfere with transmission within households could prevent the dissemination of DEC, even if independent acquisition of these pathogens by children is more important.

Infantile diarrhoea should normally be managed without antimicrobials. Antimicrobial drug therapy is however recommended when diarrhoea is invasive or persistent, and concerns are increasing in studies reporting multiple antimicrobial resistance among strains of pathogenic *E. coli* [38–40]. Our data showed the presence of multidrug-resistance in 123 of 125 (98.4 %) DEC isolates recovered in this study. Recent reports point to similar trends among enteric bacteria from Nigeria and neighboring countries [41–46]. Our data suggest that, ciprofloxacin is an option for treating persistent and invasive diarrheas in our locality, but the finding that the fluoroquinolones are the only tested antimicrobial class to which susceptibility is common is a critical concern.

The observed high prevalence of multidrug-resistance in this and other studies is worrisome and reflective of selection from heavy and indiscriminate use of common antimicrobial agents [47]. While we did not test all antimicrobial options, we did include a representative from each broad antimicrobial class. In this study, resistance to sulphonamide, tetracycline, trimethoprim, ampicillin and streptomycin was most commonly seen. Only about one in two isolates was found to be resistant to nalidixic acid and chloramphenicol, while the lowest rate of resistance was to ciprofloxacin. The relatively low rate of resistance to ciprofloxacin 9 (7.2 %) observed might be due to lower selective pressure due to its relative high cost and its relatively recent introduction into Nigerian [48]. Even then, this level of resistance is still worrisome, as many strains are nalidixic acid resistant and therefore already on the route to step-wise resistance to the fluoroquinolones. Moreover, fluroquinolone treatment failure has been documented in nalidixic acid resistant strains [49–51] and the fluoroquinolones are drugs of last resort in the treatment of infectious diseases in this part of the world.

## Conclusion

In conclusion, diarrhoeagenic *E. coli* were found in cases of childhood diarrhoea as well as in apparently healthy mothers in Ile-Ife, Nigeria. STEC was the most frequently detected pathotype but ETEC and EAEC were most commonly found in both parties in mother-child pairs. In children, diarrhoeagenic *E. coli* were most commonly detected in children aged 7 to 24 months. There was a very high rate of antibacterial resistance in diarrhoeagenic *Escherichia coli* to antibiotics that are commonly employed to treat infections with the exception of ciprofloxacin. This study highlights the importance *E. coli* pathotypes as agents of diarrhoea and points to the possibility of person-to-person transmission in their spread. Because DEC pathogens were isolated in apparently healthy mothers, the data suggest that household members such as mothers can serve as asymptomatic reservoirs for strains that are potentially pathogenic in young children. In a small number of instances, apparently identical DEC isolates were recovered from mother-child pairs. Both these findings points to a need for improved sanitation and hand washing interventions in the home as well as the development of vaccines that can protect weaned children from the most common pathotypes. Furthermore, antimicrobial stewardship programs that impact community use of these drugs are needed to contain antimicrobial resistance.

## Abbreviations

DEAC: Diffusely adherent *E. coli*
DEC: Diarrhoeagenic *E. col*
EAEC: Enteroaggregative *E. coli*
EHEC: Enterohemorrhagic *E. coli*
EIEC: Enteroinvasive *E. coli*
EPEC: Enteropathogenic *E. coli*
ETEC: Enterotoxigenic *E. coli*
PCR-RFLP: PCR-Restriction fragment length polymorphisms
STEC: Shiga toxin producing *E. coli*
